# Association between serum neurofilament light chain levels and chronic kidney disease: a cross-sectional population-based study from the National Health and Nutrition Examination Survey (2013–2014 cycle)

**DOI:** 10.1080/0886022X.2024.2427178

**Published:** 2024-11-24

**Authors:** Jian-De Lu, Kaisaierjiang Kadier, Diliyaer Dilixiati, Bingzhang Qiao, Renaguli Nuer, Abudureheman Zebibula, Mulati Rexiati, Kai Li, ShuiXue Li

**Affiliations:** a Graduate School of Xinjiang Medical University, Urumqi, China; b Department of General Surgery, Children’s Hospital of Xinjiang Uygur Autonomous Region, Xinjiang Hospital of Beijing Children’s Hospital, Urumqi, China; c Department of Cardiology, First Affiliated Hospital of Xinjiang Medical University, Urumqi, China; d Department of Urology, First Affiliated Hospital of Xinjiang Medical University, Urumqi, China; e Department of Urology, Children’s Hospital of Xinjiang Uygur Autonomous Region, Xinjiang Hospital of Beijing Children’s Hospital, Urumqi, China

**Keywords:** Neurofilament light chain, chronic kidney disease, estimated glomerular filtration rate, urinary albumin–creatinine ratio, NHANES

## Abstract

**Background:**

The relationships of serum neurofilament light chain (NfL) levels with chronic kidney disease (CKD) and renal function indicators remain controversial, and comprehensive studies with large sample sizes are lacking.

**Methods:**

In total, 2,051 participants aged 20 to 75 years were identified from the National Health and Nutrition Examination Survey (2013-2014 cycle). Logistic regression models were used to assess the associations between serum NfL levels and CKD, whereas multivariate linear models were used to investigate the relationships between serum NfL levels and two kidney function indicators, namely, estimated glomerular filtration rate (eGFR) and urinary albumin–creatinine ratio (UACR). Adjustments were made to account for potential confounding variables in the analysis. Subgroup analyses stratified by age and sex were conducted. When sNfL is incorporated into the model as continuous variables, a log transformation is applied.

**Results:**

The present study included a cohort of 2,051 individuals ranging in age from 20 to 75 years. After covariate adjustment, multivariable logistic regression revealed a significant association between high serum NfL levels and an increased prevalence of CKD (OR 1.60; 95% CI 1.40–1.82; *p* < 0.0001), which remained significant when analyzed by quartiles (p for trend <0.0001). There was a statistically significant inverse correlation between the serum NfL level and the eGFR (β=-6.34; 95% CI −8.32 to −4.37; *p* < 0.0001), as well as a positive correlation between the serum NfL level and the UACR (β = 84.67; 95% CI 19.52-149.83; *p* < 0.0001). Furthermore, when stratified by age, there were significant interactions of serum NfL levels with CKD, the eGFR, and the UACR (p for interaction = 0.008, 0.016, and 0.020, respectively).

**Conclusion:**

Serum NfL levels are positively associated with the prevalence of CKD and the UACR but negatively correlated with the eGFR, particularly in older patients.

## Introduction

Chronic kidney disease (CKD) refers to the long-lasting impairment of kidney structure and function, impacting overall health for a minimum duration of 3 months [1]. CKD has emerged as a significant global public health concern, afflicting an estimated 8% to 16% of the global populace, thereby imposing a substantial disease burden on society [2]. Based on the latest report, an estimated 37 million individuals, accounting for approximately 15% of U.S. adults, have CKD [3]. Elevated urinary albumin excretion, which serves as a marker of early kidney disease, has been established as a significant predictor of the progression of CKD. The random urinary albumin-to-creatinine ratio (UACR) is widely employed as an efficient and straightforward approach to assess and define albuminuria [4]. Additionally, the estimated glomerular filtration rate (eGFR), as a primary parameter for evaluating kidney function, is a reliable biological marker for CKD that allows for monitoring fluctuations in kidney function. As life expectancy continues to rise, the prevalence of age-related decline in kidney function and the occurrence of CKD also increase [5].

The serum neurofilament light chain (NfL), an integral subunit of neurofilaments primarily found within neuronal axons, plays a crucial role in indicating axonal damage, axonal loss, and neuronal death. In instances of such pathological events, NfL is released from axons into both the cerebrospinal fluid and bloodstream [6]. NfL has been extensively investigated and demonstrated to be a biomarker for a diverse range of neurological diseases, including Alzheimer’s dementia, stroke, frontotemporal dementia, and Parkinson’s disease, which have neurodegenerative, inflammatory, traumatic, and vascular origins [7]. In addition, NfL is not exclusively limited to neurological diseases as a biomarker, as higher serum levels of NfL have also been observed in older individuals and those with cardiovascular conditions [8]. With the advent of new technologies, such as single-molecule arrays (SiMoA°), the detection of proteins in cerebrospinal fluid in blood has increased, and an increasing number of neurological biomarkers are emerging. Recent studies have shown that NfL can serve as a biomarker for predicting the severity of chronic neurodegenerative diseases, monitoring treatment progress, and determining patient prognosis [9]. This evidence supports the notion that NfL elevation is nonspecific and not solely indicative of neurological diseases. The association of elevated serum NfL levels with older populations can be attributed to various underlying mechanisms, with declining renal function being recognized as one of the primary factors contributing to this relationship [10]. In a previous study examining a cohort of 43 healthy older individuals and 188 older diabetic patients, a significant positive correlation was identified between blood NfL levels and serum creatinine levels in both groups [11]. In addition, several studies have demonstrated a substantial correlation between renal function and various neurodegenerative disorders, including cognitive decline, Parkinson’s disease, and multiple sclerosis, all of which have the potential to induce axonal injury [12]. Ciardullo et al. [13] investigated the intricate relationship between NfL levels and all-cause mortality, integrating the eGFR as a covariate in both baseline and multivariable Cox proportional hazard models. Similarly, Van der Plas et al. [10] compared minors afflicted by kidney structural anomalies and CKD with a control cohort aged 6-16 years, revealing a correlation between NfL concentrations and deteriorating renal function in CKD individuals.

The present study aimed to explore the potential correlation between serum NfL levels and CKD, as well as two distinct indicators of renal function, *via* a comprehensive analysis encompassing noninstitutionalized adults from a wide-ranging, population-based perspective. The present study analyzed data derived from the National Health and Nutrition Examination Survey (NHANES) of the 2013-2014 cycle.

## Methods

### Study population and data collection

Data was extracted from the NHANES 2013-2014 cycle, as it was the only cycle that encompassed the factors of interest for the present study. The NHANES is a recurring population-based database that is compiled in 2-year intervals by the National Center for Health Statistics (NCHS), which is a part of the Centers for Disease Control and Prevention (CDC) in the United States. The NHANES uses a complex, multistage, stratified, clustered probability design to create a dataset representative of noninstitutionalized U.S. residents [14]. All participants provided written informed consent, and the NHANES was approved by the NCHS Ethics Review Board. In addition, the present study was performed according to the Strengthening the Reporting of Observational Studies in Epidemiology (STROBE) guidelines [15] for reporting cross-sectional studies, and it was conducted in accordance with the principles of the Declaration of Helsinki. A comprehensive description of the STROBE guidelines is provided in Supplementary Material 1.

**Figure 1.**
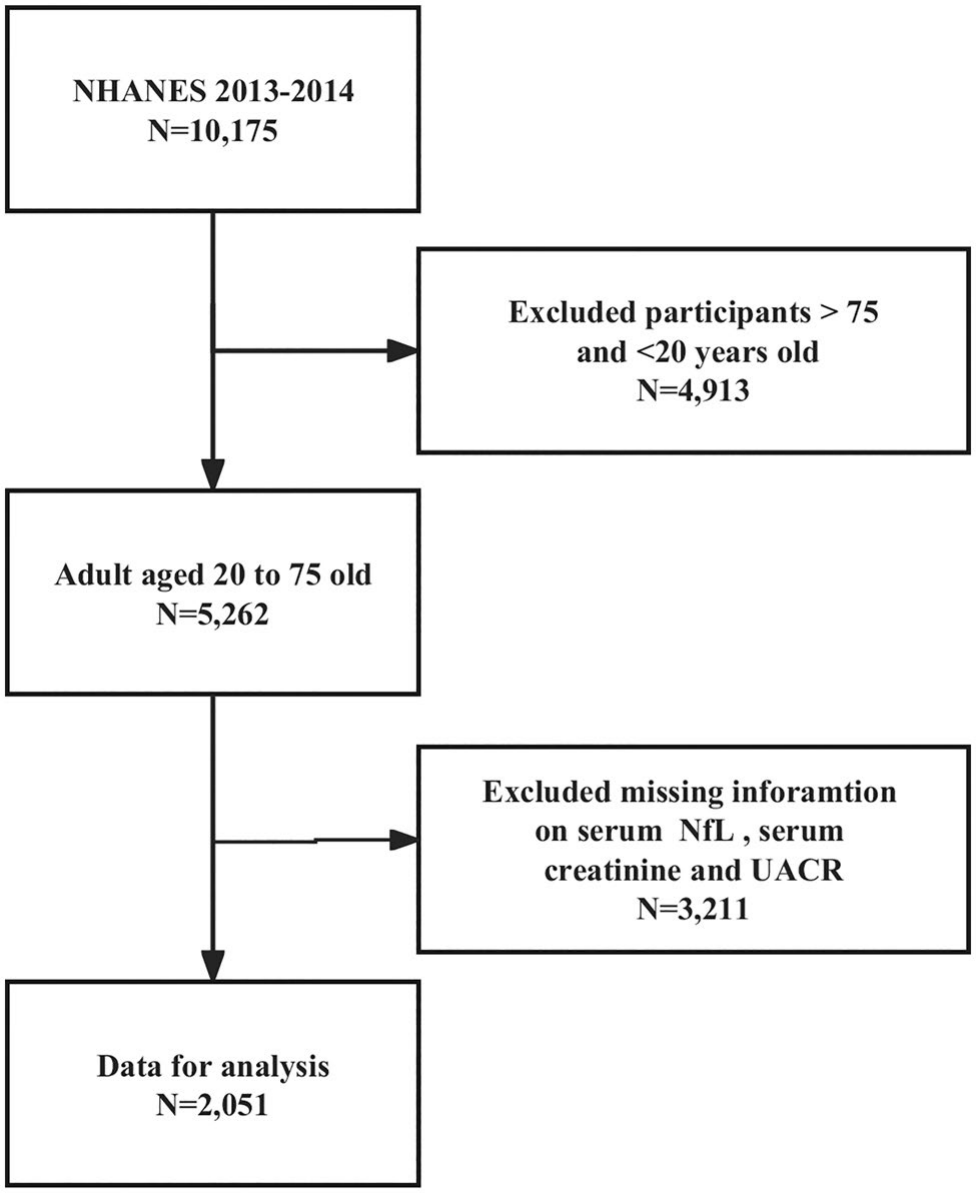
Flow chart of eligible National Health and Nutrition Examination Survey (NHANES) participants included in the present study.

All participants aged 20 years or older with complete information on serum NfL, serum creatinine levels, and the UACR from the NHANES 2013-2014 cycle were included in the present study. The NHANES 2013-2014 cycle initially consisted of 10,175 participants. To ensure data integrity and accuracy, the present study excluded participants younger than 20 years or older than 75 years (*n* = 4,913), as well as individuals with missing data for serum NfL (*n* = 3,191), serum creatinine levels, and the UACR (*n* = 20). The present study included a total of 2,051 participants. The flow diagram of sample collection is shown in Figure 1.

### Serum neurofilament light chain measurement

Serum neurofilament light chain measurements were obtained from the laboratory data in the NHANES. Serum samples obtained from a subset of NHANES 2013-2014 participants aged 20 to 75 years were included in the present study. Only those individuals who provided informed consent for the future utilization of their samples and possessed stored surplus or pristine serum samples were deemed eligible for the analysis. The samples were incubated with AE-labelled antibodies specific to the NfL antigen. Subsequently, paramagnetic particles (PMPs) coated with capture antibodies were added to the sample, facilitating the formation of antigen complexes that were simultaneously bound to AE-labelled antibodies and PMPs. Acid and base reagents were then added to initiate the process of chemiluminescence, and the subsequent emission of light was then quantitatively measured. The method demonstrated a broad detection range, ranging from 3.9 to 500 pg/mL, highlighting its capacity to precisely quantify target analytes. This approach offers several notable advantages, such as high quantum yield, rapid kinetics, excellent repeatability, hydrophilicity, hydrolysability, and stability, making it a highly favorable choice for analytical applications. For a comprehensive understanding of the serum NfL quantification procedure and the associated analytical methods employed, a thorough and detailed description can be found on the official website (http://www.cdc.gov/nchs/nhanes.htm).

### Outcome assessment

The outcomes of this study included CKD and two continuous variables, namely, eGFR and UACR, which are indicators of renal function. Serum creatinine levels were assessed *via* the Jaffe rate method and calibrated *via* a standardized isotope dilution mass spectrometry reference method. Additionally, the eGFR was calculated *via* the 2009 CKD Epidemiology Collaboration equation [16], with calibrated serum creatinine serving as the primary input. Urinary ALB levels were determined by analyzing random and single voided samples from participants *via* a solid-phase fluoroimmunoassay. CKD was defined as an eGFR < 60 mL/min/1.73 m^2^ or an UACR > 30 mg/g [17].

### Covariates

Taking into account previous studies examining serum NfL levels and CKD, renal function, and NHANES data, various clinical and social factors that may serve as potential confounding variables in the present analysis were identified. Several key covariates were incorporated in the present analysis, including participant demographic characteristics [age, sex, race, ethnicity, BMI (in kg/m^2^), poverty-income ratio (PIR), and education level]. Additionally, lifestyle factors, such as physical activity, smoking status, and alcohol consumption, were considered. To ensure a comprehensive assessment, the participants’ medical history of comorbidities, namely, hypertension, diabetes, cardiovascular disorders (CVDs), and cancer, were included. A comprehensive description of the detailed information is provided in Supplementary Material 2.

### Statistical analysis

Due to the intricate sampling survey, weighted analyses were conducted in accordance with the guidelines specified by the NHANES [18]. Categorical variables are presented as percentages and were assessed *via* the Rao–Scott chi-square test for statistical significance. Weighted participant characteristics were provided across CKD participants and non-CKD participants, and the analysis of serum NfL levels utilized log-transformed continuous variables and quartiles. Weighted logistic regression models were utilized to estimate the odds ratios (ORs) and confidence intervals (CIs) pertaining to the association between the serum NfL level and CKD. Additionally, multivariate linear regression models were used to evaluate the correlations between the serum NfL concentration and the eGFR, as well as between the serum NfL concentration and the UACR. The following three distinct models were employed to investigate the potential influence of covariates on this association: Model 1, an unadjusted crude model; Model 2, adjusted for age, sex, race, and ethnicity; Model 3, Model 2 plus adjusted for BMI, PIR, education level, physical activity, smoking status, alcohol consumption, hypertension, diabetes, CVD, and cancer.

The present study employed the MissForest R software package to address the challenges posed by missing covariates. A comprehensive description of the missing covariates is provided in Supplementary Material Table 3. The efficacy of this model in handling diverse covariate types, including both categorical and continuous variables, remained evident. The performance of the model remained robust even in the presence of missing covariate data, as exemplified by the outcomes shown in Supplementary Material 3. All analyses were performed with R version 4.1.3, and a two-tailed *p* < 0.05 was considered statistically significant.

## Results

### Baseline characteristics of participants

Table 1 shows the baseline characteristics of the participants. The study included a sample of 2,051 participants, representing a population of 169,277,364 individuals, with an average age range of 20 to 75 years, consisting of 51.33% females and 48.67% males. The majority of these individuals, 64.79% non-Hispanic White and 12.05% non-Hispanic Black, had middle or high poverty-income ratios (75.35%) and had at least a high school degree (84.19%). Furthermore, participants with and without CKD differed significantly with respect to age, sex, BMI, PIR, alcohol consumption status, hypertension, diabetes, CVD, and serum NfL levels (all *p* < 0.05). Supplementary Material 4 shows the clinical and biochemical characteristics of the population, stratified into quartiles on the basis of serum NfL levels.

**Table 1.** Baseline characteristics of CKD participants and non-CKD participants in the NHANES 2013–2014.

Characters	Overall (*n* = 2,051)	Non-CKD (*n* = 1,759)	CKD (*n* = 292)	*P*-value
Age				< 0.0001
≥ 65	12.49(10.30,14.68)	10.38(8.45,12.30)	27.47(22.60,32.35)	
45-64	37.01(32.63,41.38)	36.86(34.38,39.35)	38.03(31.47,44.59)	
20-44	50.51(44.85,56.16)	52.76(49.66,55.87)	34.49(27.92,41.07)	
Sex				< 0.001
Male	48.67(42.58,54.75)	50.22(47.95,52.50)	37.62(33.58,41.66)	
Female	51.33(46.85,55.81)	49.78(47.50,52.05)	62.38(58.34,66.42)	
Race and ethnicity				0.972
Non-Hispanic White	64.79(52.17,77.41)	64.96(57.64,72.29)	63.56(55.20,71.93)	
Non-Hispanic Black	12.05(9.25,14.84)	11.89(8.90,14.88)	13.16(8.49,17.83)	
Mexican American	9.58(6.71,12.45)	9.62(6.10,13.13)	9.29(5.83,12.74)	
Other Hispanic	5.86(2.90, 8.82)	5.84(2.60,9.08)	6.00(2.39,9.61)	
Other Race (Including Multi-Racial)	7.73(5.95, 9.51)	7.69(5.54, 9.84)	7.99(4.66,11.32)	
BMI				< 0.001
≥ 30	37.68(33.39,41.98)	35.79(33.26,38.33)	51.09(45.64,56.54)	
<30	62.32(55.68,68.96)	64.21(61.67,66.74)	48.91(43.46,54.36)	
PIR				0.049
> 3.5	40.33(30.87,49.79)	41.72(35.36,48.08)	30.49(22.25,38.72)	
1.3-3.5	35.02(31.82,38.22)	34.52(31.55,37.49)	38.55(30.52,46.58)	
< 1.3	24.65(19.31,29.98)	23.76(17.92,29.59)	30.96(20.15,41.78)	
Education level				0.091
Above high school	64.15(53.99,74.30)	65.05(59.80,70.31)	57.73(50.02,65.44)	
High school	20.04(17.19,22.90)	19.67(16.79,22.55)	22.66(16.87,28.46)	
Less than high school	15.81(12.74,18.88)	15.27(11.65,18.89)	19.61(14.36,24.85)	
Smoking status				0.148
Never	56.59(49.06,64.12)	57.69(53.20,62.18)	48.76(39.02,58.50)	
Former	22.15(17.80,26.50)	21.51(18.47,24.55)	26.69(19.98,33.40)	
Now	21.26(17.17,25.35)	20.80(16.34,25.25)	24.55(16.83,32.26)	
Alcohol consumption status				0.008
Never	11.52(7.17,15.87)	11.58(6.91,16.25)	11.08(6.26,15.90)	
Former	12.21(10.39,14.03)	10.79(8.66,12.92)	22.30(18.20,26.40)	
Mild	33.70(27.32,40.08)	34.17(29.05,39.28)	30.36(21.37,39.35)	
Heavy	42.57(37.28,47.86)	43.46(39.95,46.97)	36.25(29.84,42.66)	
Hypertension				< 0.0001
Yes	45.53(39.72,51.33)	42.17(38.66,45.68)	69.34(63.15,75.54)	
No	54.47(47.91,61.04)	57.83(54.32,61.34)	30.66(24.46,36.85)	
Diabetes				< 0.0001
Yes	10.84(9.36,12.31)	8.30(7.10, 9.49)	28.86(24.49,33.23)	
No	89.16(79.69,98.63)	91.70(90.51,92.90)	71.14(66.77,75.51)	
CVD				< 0.0001
Yes	6.80(5.01, 8.59)	5.12(3.63, 6.62)	18.67(11.84,25.50)	
No	93.20(83.44,102.96)	94.88(93.38,96.37)	81.33(74.50,88.16)	
Cancer				0.985
Yes	8.84(7.01, 10.68)	8.85(7.17,10.53)	8.78(2.24,15.32)	
No	91.16(82.15,100.17)	91.15(89.47,92.83)	91.22(84.68,97.76)	
Serum NfL levels (pg/mL)				< 0.0001
[2.8,8.2]	26.70(21.82,31.59)	27.63(24.20,31.06)	20.16(14.61,25.70)	
(8.2,12.2]	24.69(21.09,28.29)	26.01(23.59,28.43)	15.35(10.08,20.62)	
(12.2,18.9]	24.57(20.45,28.69)	25.30(21.83,28.77)	19.40(11.98,26.83)	
(18.9,497.6]	24.03(19.95,28.12)	21.07(17.21,24.92)	45.09(37.19,52.98)	
eGFR (ml/min/1.73 m²)				< 0.0001
[8.8,82.9]	25.863(21.26,30.46)	22.80(19.72,25.88)	47.57(41.65,53.49)	
(82.9,98.4]	25.46(20.87,30.05)	27.66(24.85,30.47)	9.88(5.54,14.23)	
(98.4,111.4]	23.89(21.11,26.67)	24.33(22.41,26.25)	20.79(15.03,26.55)	
(111.4,159.9]	24.79(22.32,27.25)	25.21(21.53,28.90)	21.76(15.18,28.35)	
UACR (mg/g)				< 0.0001
[0.2,4.8]	26.92(23.00,30.84)	30.15(27.55,32.74)	4.05(1.82,6.28)	
(4.8,7.1]	26.20(22.62,29.77)	29.54(27.13,31.95)	2.49(0.99,3.98)	
(7.1,13.2]	24.15(20.79,27.52)	26.16(24.05,28.26)	9.94(5.13,14.75)	
(13.2,7142.9]	22.73(20.12,25.33)	14.16(12.24,16.07)	83.53(78.10,88.95)	

Values are weighted % (95% confidence interval). *P* values are weighted.

BMI, body mass index; PIR, poverty-income ratio; CVD, Cardiovascular disorders.

### Associations between serum neurofilament light chain levels and chronic kidney disease

Table 2 shows the associations between serum NfL levels and CKD. When serum NfL is incorporated into the model as a continuous variable, a log transformation is applied. There was a significant association between serum NfL levels as a continuous covariate and CKD in Model 1 (OR 2.16; 95% CI 1.81- 2.59; *p* < 0.0001), Model 2 (OR 1.98; 95% CI 1.60-2.46; *p* < 0.001), and Model 3 (OR 1.60; 95% CI 1.40-1.82; *p* < 0.0001). In the fully adjusted model (Model 3), when serum NfL levels were analyzed on the basis of quartiles, a significantly elevated risk of CKD (OR 1.55, 95% CI 1.03-2.33, *p* = 0.037) was identified among participants in quartile 4 compared with those in quartile 1 (p for trend <0.0001).

**Table 2.** Associations between serum neurofilament light chain levels and chronic kidney disease in NHANES 2013–2014.

Exposure	Cases (*N* = 292)	N (*N* = 2051)	Model 1 OR (95%CI)	Model 2 OR (95%CI)	Model 3 OR (95%CI)
Continuous (log-transformed)			2.16(1.81,2.59) *p* < 0.0001	1.98(1.60,2.46) *p* < 0.001	1.60(1.40,1.82) *p* < 0.0001
Q1[2.8,8.2]	49	524	Ref.	Ref.	Ref.
Q2(8.2,12.2]	43	505	0.81(0.47,1.39) *p* = 0.407	0.72(0.37,1.39) *p* = 0.254	0.71(0.38,1.32) *p* = 0.257
Q3(12.2,18.9]	66	510	1.05(0.60,1.86) *p* = 0.852	0.80(0.36,1.78) *p* = 0.499	0.70(0.35,1.39) *p* = 0.279
Q4(18.9,497.6]	134	512	2.93(2.03,4.25) *p* < 0.0001	2.10(1.26,3.49) *p* = 0.013	1.55(1.03,2.33) *p* = 0.037
P for trend			<0.0001	<0.001	<0.0001

OR, odds ratio; CI, confidence interval. Model 1, a non-adjusted crude model; Model 2, adjusted with age, gender, race and ethnicity; Model 3, Model 2 plus BMI, PIR, education level, physical activity, smoking status, alcohol consumption, hypertension, diabetes, CVD and cancer.

### Associations between serum neurofilament light chain levels and renal function indicators

Table 3 shows the associations between serum NfL levels and the eGFR and UACR, as determined *via* a covariate-adjusting model. When sNfL is incorporated into the model as continuous variables, a log transformation is applied. There was a significant inverse correlation between serum NfL levels and the eGFR, as supported by the fully adjusted model (Model 3, β −9.89; 95% CI −12.65 to −7.13; *p* < 0.0001). Moreover, participants in quartiles 2–4 presented significant negative associations between the serum NfL level and eGFR compared with those in quartile 1 (all P values for trends across quartiles <0.05). There was also a positive association between the serum NfL level and UACR (Model 3, β = 84.67; 95% CI 19.52-149.83; *p* < 0.0001). Compared with participants in Q1, there was a distinction only among participants in Q4 (Model 3, β = 60.14; 95% CI 20.96-99.32; *p* = 0.005) when considering statistical significance. However, the overall trend in quartile changes remained significant (all P values for trends across quartiles <0.05).

**Table 3.** Associations between serum neurofilament light chain levels and renal function indicators in NHANES 2013–2014.

Exposure	Model 1 β(95%CI)	Model 2 β(95%CI)	Model 3 β(95%CI)
eGFR			
Continuous (log-transformed)	−14.17(-16.30,-12.04) *p* < 0.0001	−6.61(-8.60,-4.62) *p* < 0.001	−6.34(−8.32,-4.37) *p* < 0.0001
Q1[2.8,8.2]	Ref.	Ref.	Ref.
Q2(8.2,12.2]	−9.41(-11.38, −7.44) *p* < 0.0001	−3.33(−5.71,-0.95) *p* = 0.016	−3.28(−5.29,-1.26) *p* = 0.003
Q3(12.2,18.9]	−18.12(-21.37,-14.88) *p* < 0.0001	−6.88(-10.11,-3.64) *p* = 0.003	−6.91(−9.67,-4.15) *p* < 0.0001
Q4(18.9,497.6]	−24.22(-28.04,-20.39) *p* < 0.0001	−10.31(-13.72,-6.90) *p* < 0.001	−9.89(-12.65,-7.13) *p* < 0.0001
P for trend	*p* < 0.0001	*p* < 0.001	*p* < 0.0001
UACR			
Continuous (log-transformed)	84.27(14.83,153.71) *p* = 0.021	99.30(10.86,187.73) *p* = 0.033	84.67(19.52,149.83) *p* = 0.014
Q1[2.8,8.2]	Ref.	Ref.	Ref.
Q2(8.2,12.2]	−1.04(-5.06, 2.97) *p* = 0.581	−3.42(-12.68, 5.85) *p* = 0.387	−2.87(−10.30, 4.56) *p* = 0.423
Q3(12.2,18.9]	3.66(-4.08, 11.41) *p* = 0.323	3.49(-10.97, 17.95) *p* = 0.562	−7.97(−27.30, 11.37) *p* = 0.394
Q4(18.9,497.6]	82.4(21.04,143.76) *p* = 0.013	85.45(13.59,157.31) *p* = 0.028	60.14(20.96, 99.32) *p* = 0.005
P for trend	0.01	0.019	0.005

Model 1, a non-adjusted crude model; Model 2, adjusted with age, gender, race and ethnicity; Model 3, Model 2 plus BMI, PIR, education level, physical activity, smoking status, alcohol consumption, hypertension, diabetes, CVD and cancer.

### Subgroup analyses

Table 4 shows the age- and sex-stratified associations between log-transformed serum NfL levels and the prevalence rate of CKD. Significant interactions were detected among the age subgroups (p for interaction = 0.008), whereas no significant interactions were detected among the sex subgroups (p for interaction = 0.073). No statistically significant difference in the prevalence of CKD was observed among individuals aged 20-39 years. However, the associations between serum NfL levels and CKD were more pronounced in the 40–59-year-old age group (OR = 1.69, 95% CI: 1.15, 2.48; *p* = 0.011) and the 60–75-year-old age group (OR = 5.97, 95% CI: 2.41, 14.82; *p* < 0.001).

**Table 4.** Associations between serum levels of neurofilament light chain and chronic kidney disease, as well as indicators of renal function across various subgroups.

Subgroups	CKD OR(95%CI)	*P* value for interaction	eGFR β(95%CI)	*P* value for interaction	UACR β(95%CI)	*P* value for interaction
Sex		0.073		0.209		0.091
Male	1.67(1.20,2.34) *p* = 0.005		−5.76(−7.68,-3.84) *p* < 0.0001		127.60(17.86,237.33) *p* = 0.026	
Female	1.65(1.36,2.01) *p* < 0.0001		−6.97(−9.70,-4.25) *p* < 0.0001		37.78(7.65,67.90) *p* = 0.017	
Age		0.008		0.016		0.021
20–39	1.09(0.62,1.94) 0.749		−5.54(-7.83,-3.24) *p* < 0.001		23.64(−0.17,47.45) *p* = 0.051	
40–59	1.69(1.15, 2.48) 0.011		−6.36(−8.83,-3.90) *p* < 0.0001		189.62(52.02,327.21) *p* = 0.010	
60–75	5.97(2.41,14.82) <0.001		−10.44(-14.88,-5.99) *p* < 0.001		26.47(2.04,50.90) *p* = 0.036	

Each stratification factor was adjusted for age, gender, race and ethnicity, BMI, PIR, education level, physical activity, smoking status, alcohol consumption, hypertension, diabetes, CVD and cancer. OR, odds ratio; 95% CI, 95% confidence interval. When sNfL is incorporated into the model as continuous variables, a log transformation is applied.

Table 4 also shows the associations of the serum log-transformed NfL level with the eGFR and UACR within specific age and sex subgroups. Consistent with previous subgroup analyses of CKD patients, significant interactions were detected within the age subgroups (p for interaction = 0.016 and 0.020, respectively), whereas no significant interactions were detected within the sex subgroups.

## Discussion

The present cross-sectional study of nationally representative data from the USA analyzed data from the NHANES (2013-2014) database, which included 2,051 participants aged 20 to 75 years. In addition, adhering rigorously to international standards [18], the present study defined CKD by using two diagnostic indices, the eGFR and UACR, as the primary subjects of observation. The present study revealed a significant association between high serum NfL levels and an increased prevalence of CKD. Analysis of serum NfL levels across quartiles indicated a substantially greater risk of CKD among participants in quartile 4 than among those in quartile 1. Moreover, an inverse relationship was detected between the serum NfL level and indicators of renal function, specifically a significant inverse correlation between the serum NfL level and eGFR, while a positive association between the serum NfL level and UACR was observed. Furthermore, associations of serum NfL levels with CKD, the eGFR, and the UACR, which were stratified according to age group.

Van der Plas et al. investigated the relationship between plasma NfL concentrations and declining renal function in a cohort of 16 children diagnosed with CKD resulting from congenital renal malformations, and they reported that plasma NfL levels in children with CKD increase alongside the progressive decline in renal function with increasing age [10]. Nevertheless, the scope of the study by Van der Plas et al. is confined to minors, necessitating further validation of its implications for adults within an adult population, which was addressed by the present study. Liu [19] et al. recently investigated the relationship between sNfL and UACR, revealing a J-shaped correlation between the two using RCS curves and identifying the key inflection point of the sNfL. However, unlike their study, our primary observation involves the use of a logistic regression model to assess the relationship between serum NfL levels and CKD, while our secondary observation employs a multivariable linear model to examine the relationship between serum NfL levels and two renal function indicators: eGFR and UACR. From a concluding perspective, the aforementioned study aligns fundamentally with our findings on the relationship between sNfL and UACR. Notably, in two cohort studies [20,21], one from the Shanghai Aging Study (SAS) and the other from the Gothenburg H70 Birth Cohort 1944, researchers examined the relationship between renal function and serum NfL, concluding that eGFR levels were inversely correlated with plasma NfL concentrations. This finding is consistent with the conclusion of this study that plasma NfL concentrations and eGFR levels are inversely proportional. In addition, Polymeris et al. conducted a study on the older patients with atrial fibrillation, building upon the results of previous studies that examined a small sample of individuals with diabetes [10,22], and their findings revealed a substantial inverse correlation between the eGFR and serum NfL concentration [19]. Furthermore, researchers [13] have focused mainly on the relationship between NfL levels and all-cause mortality, using the eGFR as a baseline and a covariate in a multivariable Cox proportional hazards model. A significant negative correlation between the eGFR and the serum NfL concentration has been reported. However, the present study investigated the relationship between NfL levels and CKD using logistic regression models. Multivariate linear models were employed to investigate the relationships between serum NfL levels and kidney function parameters, including the eGFR and UACR. Additionally, potential confounding factors were adjusted for in the analysis, and subgroup analyses were stratified by age and sex. Furthermore, the present study extends the generalizability of this association to a large, population-based sample, representing the noninstitutionalized population. By addressing limitations in terms of age (20–75 years) and comorbidity factors, the present study provides a more comprehensive understanding.

The pathophysiological mechanism underlying the link between serum NfL levels and CKD, as well as renal function, remains elusive. Despite this, several potential mechanisms have emerged that provide indications of a plausible relationship between these variables. First, it is important to note that in individuals suffering from chronic CKD, impaired renal function leads to the accumulation of crucial molecular mediators of brain injury as the eGFR decreases [23]. This impaired renal function also predisposes these individuals to a higher incidence of cerebrovascular disease [24]. Cerebrovascular disease, in turn, induces nerve damage, resulting in elevated levels of serum NfL [25]. Second, recognizing the substantial influence of common disease factors on this phenomenon is essential. Hypertension, as an example, disrupts the self-regulatory mechanisms of the brain and is commonly linked to chronic hypoperfusion, cerebral ischemia, and lacunar infarction. This association is predominantly attributed to hypertension-induced activation of the renal renin–angiotensin system (RAS), disruption of the blood–brain barrier, and neuronal damage triggered by endothelin and ischemic events [26,27]. Moreover, hypertension not only contributes to a decline in renal function but also accelerates the development of renal dysfunction. Additionally, diabetes-induced peripheral nerve injury can lead to an elevation in serum NfL levels *via* mechanisms intricately connected to nerve damage. Third, an elevation in serum NfL levels does not necessarily imply the existence of neurodegenerative disorders or other coexisting medical conditions. Age, a significant contributing factor to increased NfL levels, may be associated with an increased likelihood of CKD development. As mentioned earlier, Van Der Plas et al. reported a significant positive association between age and NfL in pediatric CKD patients with fewer comorbidities [10].

In the WhiteHall II cohort study, which involved a continuous analysis of eGFR measurements over a period of 10 years, a correlation was discovered between dementia and decreased eGFR levels; this association demonstrated a progressive increase as the eGFR decreased further [28]. In line with previous findings in older adult populations, the present study revealed a negative correlation between eGFR and serum NfL levels among individuals from diverse age groups. The present study also demonstrated a strong association (steep slope) between the serum NfL concentration and eGFR, particularly at lower NfL concentrations. In contrast to previously reported results [19], the present findings revealed a significant age interaction that was further amplified by the broad age range encompassed in the present study. The age-related elevation in serum NfL levels may be primarily attributed to the physiological decline in cerebrospinal fluid turnover, as well as the heightened axonal damage that occurs with advancing age [29]. In addition, the calculation of eGFR relies on calibrated serum creatinine levels, causing variations in eGFR values on the basis of individual muscle mass. Specifically, individuals with greater muscle mass may have lower eGFRs [30]. The present findings supported the hypothesis that individuals characterized by diminished muscle mass (advanced age and neurological impairment) are at increased risk of experiencing both a reduced eGFR and CKD, as evidenced by decreased serum NfL levels. Several cohort studies conducted in both US and Korean populations have consistently demonstrated a significant association between sarcopenia and the stage of kidney disease, as well as the severity of CKD, indicating an elevated risk of sarcopenia with advancing stages of CKD [31,32]. Moreover, Saak et al. [33] investigated the relationships among myodystrophy, myotonic dystrophy, mitochondrial disease genes, neuronal injuries, and serum NfL levels, and they demonstrated that NfL levels can serve as a valuable indicator for detecting and tracking neuronal injuries caused by myopathy, including in individuals without apparent central nervous system involvement of clinical significance. The outcomes of these investigations further substantiate the precision of the present findings and assertions.

This study investigates the correlation between serum NfL levels and UACR. Inflammation and oxidative stress may play pivotal roles in the association between these two factors. The pathophysiology of proteinuria primarily involves disruption of the glomerular filtration barrier, increased permeability, and dysfunction in protein reabsorption subsequent to tubular epithelial cell injury. Notably, glomerular dysfunction has emerged as the predominant underlying cause of proteinuria [34]. In patients with impaired renal function, elevated tumor necrosis factor-alpha (TNF-α) contributes to glomerulosclerosis and a decline in renal function *via* enhancement of glomerular oxidative stress and promotion of glomerular injury and proteinuria by increasing the infiltration of mononuclear cells into the glomerulus [35]. The mechanisms underlying oxidative stress in CKD involve two key factors, namely, impaired mitochondrial function and the activation of polymorphonuclear leukocytes by uremic toxins. These factors contribute to the generation of reactive oxygen species through two distinct pathways, ultimately driving oxidative processes and promoting inflammatory responses [36]. As systemic inflammation persists and intensifies, the integrity of the vascular blood–brain barrier may be compromised, resulting in increased permeability to solutes [37]. As renal function decreases and CKD progresses, the clearance of uremic toxins becomes compromised, enabling their translocation across an impaired blood–brain barrier [38]. In parallel, within the context of neurological disease and nerve injury, the spread of inflammatory factors extends beyond the blood–brain barrier, resulting in renal dysfunction. Furthermore, the presence of reactive oxygen species can contribute to the manifestation of proteinuria by inflicting damage to glomerular podocytes. Using an animal model of CKD, a previous study has reported a profound disruption in the activities of key antioxidant enzymes, namely, superoxide dismutase and catalase, in addition to the manifestation of astrocytosis within the substantia nigra [39], highlighting how oxidative stress conditions in CKD may correlate with neuronal damage and serum NfL levels.

A comprehensive study has investigated the alterations in plasma markers, including neuron-specific enolase, brain-derived neurotrophic factor, and NfL, as well as levels of circulating plasma extracellular vesicles, among recipients who underwent renal transplantation, both during the transplantation procedure and two years after surgical intervention. In addition to a decrease in neuron-specific enolase and an increase in brain-derived neurotrophic factor, a concomitant increase in NfL levels was detected. The author postulated that the patients included in the study exhibited a phenotype indicative of premature vascular aging and proposed that the heightened occurrence of cardiovascular complications could account for the manifestation of this phenomenon [40]. In contrast to the findings of the aforementioned study, Blankenship [41] et al. recently assessed Alzheimer’s disease (AD) biomarkers in 46 CKD patients before kidney transplantation (KT), at 12 weeks post-KT, and at 12 months post-KT, along with baseline measurements in 13 non-CKD control participants. The study evaluated eGFR and cognitive function, and found that the improvement in renal function following KT led to a reversal of elevated AD blood biomarkers in CKD patients.

A strength of the present study was the utilization of a population-representative dataset. The present study not only enhanced the generalizability of this association to a large, population-based sample that accurately represents the noninstitutionalized population but also considered age and comorbid factors to provide a comprehensive understanding. Moreover, the present study investigated the correlation between serum NfL levels and urinary indicators for UACR, thereby expanding the scope of this association. However, the present study had several limitations. First, it is essential to acknowledge that the NHANES study, which is an observational nationwide survey conducted over multiple years, may have captured diverse variables across different time periods, thereby allowing potential unmeasured confounders. While efforts were made to account for relevant covariates on the basis of previous research, some significant factors may not have been considered or quantified, potentially impacting the precision of the present findings. Second, the present analysis was limited to individuals within the United States. Although the NHANES employs weighted sampling to include ethnic minorities, the presence of known ethnic disparities in neurological impairment, kidney disease, and prognosis imposes limitations on the generalizability of the present findings to other ancestral groups. Third, because the present study was a cross-sectional study utilizing NHANES data, it can only be concluded that there is a cross-sectional association between NfL and CKD, as well a potential causal relationship possibly due to the nature of the data. In addition, as an innovative study, the present evidence does not support NfL as a pivotal indicator within clinically utilized CKD classification or observed renal function assessments. Finally, cystatin C is as effective as creatinine in evaluating the glomerular filtration rate and may be particularly suitable for the present study because of its independence from muscle mass. However, owing to the unavailability of cystatin C data in the 2013-2014 NHANES database, the present study did not analyze cystatin C.

## Conclusions

In conclusion, the present comprehensive national population-based study revealed a positive association of the serum NfL level with the prevalence of CKD and the UACR. In addition, the present study identified a negative correlation between the serum NfL level and the eGFR. The present findings further revealed that these associations are pronounced in older patients, highlighting the significance of the serum NfL level as a potential biomarker for an increased risk of CKD, a decreased eGFR, and an elevated UACR. Finally, because the findings are particularly pronounced in older patients with a greater risk of cerebrovascular disease, restoration of renal function may not effectively reverse nerve injury, leading to persistent elevation of products associated with nerve damage.

## Supplementary Material

Supplemental Material
