# Supplementary material for: Association between serum neurofilament light chain levels and chronic kidney disease: a cross-sectional population-based study from the National Health and Nutrition Examination Survey (2013–2014 cycle)
Source: Ren Fail. 2024 Nov 24;46(2):2427178. doi: 10.1080/0886022X.2024.2427178 (PMC11590190; doi:10.1080/0886022X.2024.2427178)
Supplement: Supplemental Material [file IRNF_A_2427178_SM8942.docx]

Supplementary Material

**Supplementary Material 1:** STROBE Checklist of this study.

**Supplementary Material 2:** A comprehensive description of the Covariates.

**Supplementary Material 3:** The numbers and percentages of missing covariate date.

**Supplementary Material 4:** Baseline characteristics of the study population in the NHANES 2013–2014.

Supplementary Material 1 STROBE Statement—Checklist of items that should be included in reports of ***cross-sectional studies***

|  | Item No | Recommendation |  |
| --- | --- | --- | --- |
| **Title and abstract** | 1 ✓abstract+title  ✓ statistical analysis | (*a*) Indicate the study’s design with a commonly used term in the title or the abstract |  |
|  |  | (*b*) Provide in the abstract an informative and balanced summary of what was done and what was found |  |
| Introduction | | | |
| Background/rationale | 2 ✓ introduction | Explain the scientific background and rationale for the investigation being reported |  |
| Objectives | 3 ✓ introduction | State specific objectives, including any prespecified hypotheses |  |
| Methods | | | |
| Study design | 4 ✓ Study population and data collection | Present key elements of study design early in the paper |  |
| Setting | 5 ✓Study population and data collection | Describe the setting, locations, and relevant dates, including periods of recruitment, exposure, follow-up, and data collection |  |
| Participants | 6 ✓ Study population and data collection | (*a*) Give the eligibility criteria, and the sources and methods of selection of participants |  |
| Variables | 7 ✓ Serum Neurofilament Light Chain Measurement  Outcome Assessment  Covariates | Clearly define all outcomes, exposures, predictors, potential confounders, and effect modifiers. Give diagnostic criteria, if applicable |  |
| Data sources/ measurement | 8* ✓ Serum Neurofilament Light Chain Measurement  Outcome Assessment  Covariates | For each variable of interest, give sources of data and details of methods of assessment (measurement). Describe comparability of assessment methods if there is more than one group |  |
| Bias | 9 ✓ statistical analysis | Describe any efforts to address potential sources of bias |  |
| Study size | 10 ✓ study population; statistical analysis | Explain how the study size was arrived at |  |
| Quantitative variables | 11 ✓ statistical analysis | Explain how quantitative variables were handled in the analyses. If applicable, describe which groupings were chosen and why |  |
| Statistical methods | 12 ✓ statistical analysis  ✓ statistical analysis  ✓ statistical analysis  ✓ statistical analysis  ✓ statistical analysis | (*a*) Describe all statistical methods, including those used to control for confounding |  |
|  |  | (*b*) Describe any methods used to examine subgroups and interactions |  |
|  |  | (*c*) Explain how missing data were addressed |  |
|  |  | (*d*) If applicable, describe analytical methods taking account of sampling strategy |  |
|  |  | (*e*) Describe any sensitivity analyses |  |
| Results | | | |
| Participants | 13* ✓Baseline characteristics of participants  ✓Figure 1 | (a) Report numbers of individuals at each stage of study—eg numbers potentially eligible, examined for eligibility, confirmed eligible, included in the study, completing follow-up, and analysed |  |
|  |  | (b) Give reasons for non-participation at each stage |  |
|  |  | (c) Consider use of a flow diagram |  |
| Descriptive data | 14* ✓ Table 1  Baseline characteristics of participants | (a) Give characteristics of study participants (eg demographic, clinical, social) and information on exposures and potential confounders |  |
|  |  | (b) Indicate number of participants with missing data for each variable of interest |  |
| Outcome data | 15* ✓ Association between serum neurofilament light chain levels and chronic kidney disease  ✓Association between serum neurofilament light chain levels and renal function indicators | Report numbers of outcome events or summary measures |  |
| Main results | 16 ✓ Table 2; Table 3; Table 4  ✓ Association between serum neurofilament light chain levels and chronic kidney disease | (*a*) Give unadjusted estimates and, if applicable, confounder-adjusted estimates and their precision (eg, 95% confidence interval). Make clear which confounders were adjusted for and why they were included |  |
|  |  | (*b*) Report category boundaries when continuous variables were categorized |  |
|  |  | (*c*) If relevant, consider translating estimates of relative risk into absolute risk for a meaningful time period |  |
| Other analyses | 17 ✓ Subgroup analyses  Table 4  Supplementary Material 1-4 | Report other analyses done—eg analyses of subgroups and interactions, and sensitivity analyses |  |
| Discussion | | | |
| Key results | 18 ✓ discussion – 1^st^ paragraph | Summarise key results with reference to study objectives |  |
| Limitations | 19 ✓ discussion – 7^th^ paragraph | Discuss limitations of the study, taking into account sources of potential bias or imprecision. Discuss both direction and magnitude of any potential bias |  |
| Interpretation | 20 ✓ discussion – 2^nd^-5^th^ paragraphs | Give a cautious overall interpretation of results considering objectives, limitations, multiplicity of analyses, results from similar studies, and other relevant evidence |  |
| Generalisability | 21 ✓ discussion – 6^th^ paragraphs | Discuss the generalisability (external validity) of the study results |  |
| Other information | | | |
| Funding | 22 ✓Funding Sources | Give the source of funding and the role of the funders for the present study and, if applicable, for the original study on which the present article is based |  |

*Give information separately for exposed and unexposed groups.

**Note:** An Explanation and Elaboration article discusses each checklist item and gives methodological background and published examples of transparent reporting. The STROBE checklist is best used in conjunction with this article (freely available on the Web sites of PLoS Medicine at http://www.plosmedicine.org/, Annals of Internal Medicine at http://www.annals.org/, and Epidemiology at http://www.epidem.com/). Information on the STROBE Initiative is available at www.strobe-statement.org.

**Supplementary Material 2: A comprehensive description of the Covariates**

Race and ethnicity was self-reported, and participants were categorized into Mexican American, non-Hispanic white, non-Hispanic black, Hispanic and other race. BMI was calculated as weight divided by the square of height and described as underweight or normal (< 30 kg/m2), overweight or obese (≥ 30 kg/m2) . In their lifetime, individuals those who never smoked more than 100 cigarettes , those who smoked more than 100 cigarettes and had quit smoking, and those who smoked at least 100 cigarettes without quitting were defined as never smokers, former smokers, and current smokers, respectively. For alcohol use the boundary between 'yes' and 'no' is at least 12 drinks of alcohol or alcoholic beverages in the past year. Participants' education level was grouped into three categories, less than high school, high school graduate, and above high school. PIR was stratified as ≤1.3, 1.3–1.8, and >1.8, based on data from the original survey.A history of hypertension was defined as a self-reported physician diagnosis of hypertension or a mean systolic blood pressure ≥ 130 mm Hg or diastolic blood pressure ≥ 80 mm. Diabetes mellitus was defined as the use of any treatment for diabetes mellitus, diagnosis by a healthcare professional, or HbA1c level (%) > 6.5 and random blood glucose level (mmol/L) ≥ 11.1.

**Supplementary Material 3: The numbers and percentages of missing covariate date.**

| Covariate | Numbers | Percentages% |
| --- | --- | --- |
| PIR | 146 | 7.12 |
| Education | 3 | 0.15 |
| Smoking status | 1 | 0.05 |
| BMI | 12 | 0.59 |
| DM | 18 | 0.88 |
| alcohol user | 157 | 7.65 |

**Supplementary Material 4:** Baseline characteristics of quartiles based on serum NfL levels of the study population in the NHANES 2013–2014.

| **Characters** | Overall (n=2,051) | Q1 (n=524) | Q2 (n=505) | Q3 (n=510) | Q4 (n=512) | P-value |
| --- | --- | --- | --- | --- | --- | --- |
|  |  | [2.8,8.2] | (8.2,12.2] | (12.2,18.9] | (18.9,497.6] |  |
| **Age** |  |  |  |  |  | < 0.0001 |
| 20-44 | 50.51(44.85,56.16) | 84.10(81.23,86.97) | 55.07(50.13,60.01) | 34.04(28.62,39.45) | 25.33(21.20,29.45) |  |
| 45-64 | 37.01(32.63,41.38) | 15.84(12.94,18.74) | 38.61(34.63,42.60) | 49.53(45.27,53.79) | 46.07(41.20,50.93) |  |
| ≥ 65 | 12.49(10.30,14.68) | 0.06(-0.05, 0.17) | 6.32( 3.07, 9.57) | 16.43(13.14,19.72) | 28.61(23.20,34.01) |  |
| **Sex** |  |  |  |  |  | < 0.001 |
| Male | 48.67(42.58,54.75) | 42.41(39.11,45.71) | 49.96(45.42,54.50) | 48.33(45.34,51.32) | 54.63(51.84,57.41) |  |
| Female | 51.33(46.85,55.81) | 57.59(54.29,60.89) | 50.04(45.50,54.58) | 51.67(48.68,54.66) | 45.37(42.59,48.16) |  |
| **Race and ethnicity** |  |  |  |  |  | 0.001 |
| Non-Hispanic White | 64.79(52.17,77.41) | 54.01(45.82,62.19) | 62.04(52.78,71.31) | 71.72(62.58,80.85) | 72.52(64.26,80.78) |  |
| Non-Hispanic Black | 12.05( 9.25,14.84) | 14.04(8.97,19.10) | 14.61(9.82,19.40) | 8.20(5.22,11.18) | 11.13(7.95,14.32) |  |
| Mexican American | 9.58( 6.71,12.45) | 15.67(11.42,19.93) | 9.18( 5.29,13.07) | 6.00( 2.47, 9.53) | 6.87( 2.99,10.75) |  |
| Other Race (Including Multi-Racial) | 7.73( 5.95, 9.51) | 9.48(6.34,12.62) | 7.71(4.95,10.47) | 7.69(4.39,10.99) | 5.84(3.01, 8.67) |  |
| Other Hispanic | 5.86( 2.90, 8.82) | 6.80(2.67,10.93) | 6.46(2.88,10.04) | 6.40(2.45,10.35) | 3.64(0.13, 7.15) |  |
| **BMI** |  |  |  |  |  | < 0.001 |
| <30 | 62.32(55.68,68.96) | 55.50(51.05,59.96) | 67.18(63.71,70.65) | 69.44(64.49,74.40) | 57.61(53.13,62.09) |  |
| ≥ 30 | 37.68(33.39,41.98) | 44.50(40.04,48.95) | 32.82(29.35,36.29) | 30.56(25.60,35.51) | 42.39(37.91,46.87) |  |
| **PIR** |  |  |  |  |  | 0.013 |
| < 1.3 | 24.65(19.31,29.98) | 26.31(21.46,31.16) | 25.07(19.93,30.21) | 23.86(15.29,32.43) | 23.18(13.86,32.50) |  |
| 1.3-3.5 | 35.02(31.82,38.22) | 42.08(36.04,48.13) | 30.59(25.89,35.29) | 30.11(24.98,35.23) | 36.74(31.57,41.91) |  |
| > 3.5 | 40.33(30.87,49.79) | 31.61(24.94,38.27) | 44.34(38.11,50.57) | 46.04(38.06,54.01) | 40.08(29.97,50.19) |  |
| **Education level** |  |  |  |  |  | 0.683 |
| Above high school | 64.15(53.99,74.30) | 63.98(58.21,69.76) | 62.61(56.39,68.82) | 66.47(59.92,73.02) | 63.54(56.25,70.84) |  |
| High school | 20.04(17.19,22.90) | 18.29(12.59,23.99) | 22.03(17.13,26.92) | 19.30(15.86,22.75) | 20.70(15.96,25.45) |  |
| Less than high school | 15.81(12.74,18.88) | 17.72(13.80,21.64) | 15.37(11.39,19.34) | 14.23( 9.96,18.50) | 15.75( 9.09,22.41) |  |
| **Smoking status** |  |  |  |  |  | 0.025 |
| Never | 56.59(49.06,64.12) | 64.16(60.40,67.92) | 58.93(51.63,66.22) | 52.09(46.86,57.32) | 50.38(43.19,57.58) |  |
| Former | 22.15(17.80,26.50) | 15.70(12.91,18.50) | 21.58(16.32,26.83) | 25.19(21.10,29.29) | 26.79(20.26,33.32) |  |
| Now | 21.26(17.17,25.35) | 20.14(16.86,23.42) | 19.50(14.09,24.90) | 22.72(16.65,28.78) | 22.83(14.49,31.16) |  |
| **Alcohol consumption status** |  |  |  |  |  | 0.007 |
| never | 11.52( 7.17,15.87) | 13.16(8.56,17.76) | 9.86(5.47,14.26) | 11.85(5.82,17.89) | 11.05(6.61,15.49) |  |
| Former | 12.21(10.39,14.03) | 7.51( 4.96,10.05) | 10.08( 7.00,13.16) | 13.87(10.70,17.03) | 17.93(14.51,21.35) |  |
| Mild | 33.70(27.32,40.08) | 29.48(24.97,33.99) | 36.44(30.56,42.33) | 34.68(26.47,42.89) | 34.56(24.94,44.18) |  |
| heavy | 42.57(37.28,47.86) | 49.85(44.49,55.21) | 43.61(38.75,48.47) | 39.60(34.26,44.93) | 36.46(30.17,42.75) |  |
| **Hypertension** |  |  |  |  |  | < 0.0001 |
| Yes | 45.53(39.72,51.33) | 26.70(21.59,31.82) | 40.70(34.07,47.33) | 51.76(44.68,58.84) | 65.03(58.13,71.93) |  |
| No | 54.47(47.91,61.04) | 73.30(68.18,78.41) | 59.30(52.67,65.93) | 48.24(41.16,55.32) | 34.97(28.07,41.87) |  |
| **Diabetes** |  |  |  |  |  | < 0.0001 |
| Yes | 10.84( 9.36,12.31) | 3.95( 2.78, 5.12) | 6.76( 4.01, 9.51) | 12.43( 9.42,15.44) | 21.05(17.09,25.01) |  |
| No | 89.16(79.69,98.63) | 96.05(94.88,97.22) | 93.24(90.49,95.99) | 87.57(84.56,90.58) | 78.95(74.99,82.91) |  |
| **CVD** |  |  |  |  |  | < 0.0001 |
| Yes | 6.80( 5.01, 8.59) | 0.75(-0.04, 1.54) | 5.06( 2.42, 7.70) | 7.43( 4.36,10.51) | 14.66( 9.08,20.24) |  |
| No | 93.20(83.44,102.96) | 99.25(98.46,100.04) | 94.94(92.30, 97.58) | 92.57(89.49, 95.64) | 85.34(79.76, 90.92) |  |
| **Cancer** |  |  |  |  |  | 0.001 |
| Yes | 8.84( 7.01, 10.68) | 3.59(2.21, 4.97) | 8.42(5.66,11.19) | 11.46(7.51,15.41) | 12.44(9.35,15.54) |  |
| No | 91.16(82.15,100.17) | 96.41(95.03,97.79) | 91.58(88.81,94.34) | 88.54(84.59,92.49) | 87.56(84.46,90.65) |  |
| **CKD** |  |  |  |  |  | < 0.0001 |
| Yes | 12.36(10.23,14.48) | 9.33( 6.22,12.44) | 7.68( 4.76,10.60) | 9.76( 5.95,13.56) | 23.18(20.07,26.30) |  |
| No | 87.64(78.12,97.16) | 90.67(87.56,93.78) | 92.32(89.40,95.24) | 90.24(86.44,94.05) | 76.82(73.70,79.93) |  |
| **eGFR (ml/min/1.73 m²)** |  |  |  |  |  | < 0.0001 |
| [8.5,82.9] | 25.86(21.26,30.46) | 7.94( 4.85,11.03) | 17.33(12.82,21.85) | 35.29(28.04,42.54) | 44.90(37.86,51.95) |  |
| (82.9,98.4] | 25.46(20.87,30.05) | 14.36(11.37,17.34) | 28.73(24.68,32.78) | 30.77(24.98,36.56) | 29.01(25.15,32.86) |  |
| (98.4,111.4] | 23.89(21.11,26.67) | 28.45(25.31,31.58) | 31.03(26.90,35.16) | 21.32(16.67,25.98) | 14.12( 8.89,19.34) |  |
| (111.4,159.9] | 24.79(22.32,27.25) | 49.26(43.89,54.63) | 22.91(17.15,28.66) | 12.61( 7.62,17.61) | 11.98( 8.90,15.05) |  |
| **UACR (mg/g)** |  |  |  |  |  | 0.006 |
| [0.2,4.8] | 26.92(23.00,30.84) | 30.02(23.42,36.62) | 30.27(25.45,35.09) | 25.51(21.82,29.20) | 21.49(17.01,25.97) |  |
| (4.8,7.1] | 26.20(22.62,29.77) | 26.51(22.22,30.81) | 26.07(20.23,31.91) | 30.65(27.15,34.15) | 21.41(18.68,24.15) |  |
| (7.1,13.2] | 24.15(20.79,27.52) | 21.55(17.41,25.69) | 23.39(19.60,27.19) | 26.13(22.08,30.18) | 25.80(22.26,29.35) |  |
| (13.2,7142.9] | 22.73(20.12,25.33) | 21.91(18.18,25.65) | 20.27(14.21,26.32) | 17.71(13.91,21.52) | 31.29(26.86,35.73) |  |

Values are weighted % (95% confidence interval). P values are weighted.

BMI, body mass index;PIR,poverty-income ratio; CVD,Cardiovascular disorders.
